# The Association between Diurnal Sleep Patterns and Emotions in Infants and Toddlers Attending Nursery

**DOI:** 10.3390/brainsci10110891

**Published:** 2020-11-22

**Authors:** Valeria Bacaro, Bernd Feige, Fee Benz, Anna F. Johann, Paola De Bartolo, Alessandra Devoto, Caterina Lombardo, Dieter Riemann, Chiara Baglioni

**Affiliations:** 1 Department of Human Sciences, University of Rome Guglielmo Marconi, Via Plinio, 44, 00193 Roma, Italy; v.bacaro@unimarconi.it (V.B.); p.debartolo@unimarconi.it (P.D.B.); 2 Department of Psychiatry and Psychotherapy. Faculty of Medicine. Medical Center—University of Freiburg, University of Freiburg, 79085 Freiburg im Breisgau, Germany; bernd.feige@uniklinik-freiburg.de (B.F.); fee.benz@uniklinik-freiburg.de (F.B.); anna.johann@uniklinik-freiburg.de (A.F.J.); dieter.riemann@uniklinik-freiburg.de (D.R.); 3 Medical Psychology and Medical Sociology, Faculty of Medicine, University of Freiburg, 79085 Freiburg im Breisgau, Germany; 4 Centre for Sleep Medicine, 00147 Rome, Italy; alessandra.devoto@uniroma1.it; 5 Department of Psychology, Sapienza University of Rome, Piazzale Aldo Moro, 5, 00185 Roma, Italy; caterina.lombardo@uniroma1.it

**Keywords:** sleep, positive emotions, negative emotions, infancy, toddlerhood, nursery, diurnal sleep

## Abstract

Background: Childcare programs often include mandatory naptime during the day. Loss of daytime sleep could lead to a moderate-to-large decrease in self-regulation, emotion processing, and learning in early childhood. Nevertheless, daytime sleep has been less accurately studied than nighttime sleep. This study aims to explore the relationship between diurnal sleep habits in nursery settings, nocturnal sleep quality, and post-nap emotional intensity in infants and toddlers. Methods: Data of 92 children (52 girls, 40 boys) aged 6 to 36 months were obtained. Sleep habits as well as positive and negative emotions were monitored by educators during nursery times through a sleep and emotion diary for two weeks. Results: Explorative analyses showed that diurnal sleep hours decreased across age groups (except for females aged 25–36 months) and that all age groups had a lower amount of nocturnal sleep than is recommended by the National Sleep Foundation. Partial correlation analysis showed significant correlation between daytime sleep onset latency and positive emotions. Mediation analyses showed that daytime napping is relevant for emotional functioning independently of nocturnal sleep quality. Conclusions: Daytime sleep in early childhood seems to be linked to the management of positive and negative emotions and could play a role in healthy development of emotional processes.

## 1. Introduction

Sleep patterns evolve rapidly over the first 3 years of life. Newborns spend as much as 80% of their day sleeping, and most toddlers spend half or more of their day asleep [1,2]. By six months of age, the wakeful period consolidates during the daytime, but the practice of taking at least one nap during the day persists during toddlerhood [3,4,5]. Healthy sleep is associated with key developmental factors, including general psychological functioning [6], cognitive performance [7], and overall developmental status [8]. Furthermore, poor sleep was found to be associated to social interpersonal relationship as peer acceptance, social skills, social engagement and emotions’ understanding [8]. At the same time, early social and emotional development is also considered crucial for a healthy socioemotional development [9].

### 1.1. Nocturnal Sleep and Socio-Emotional Development

In a questionnaire study, Hysing and colleagues [10] demonstrated that less total sleep, prolonged sleep onset, and frequent night awakenings corresponded to greater risk of having concurrent social–emotional problems in toddlerhood. Moreover, Mindell and colleagues [11] conducted a longitudinal study of toddlers aged 3 to 18 months and found that sleep alterations, such as later bedtimes and less total sleep duration, appear to be associated with internalizing issues in infants and toddlers. Sleep seems to play a key role in cognitive and affective processes central to self-regulation in school-age children [12], but less is known about younger children.

### 1.2. Diurnal Sleep and Socio-Emotional Development

Daytime sleep also has a role in the healthy development in early childhood. In a systematic review, Thorpe and colleagues [13] summarized 26 articles about the effects of napping time and quality on development and health in young children. The authors found the results to be inconsistent, most probably because of the different age groups involved (0–5 years) and different napping statuses of the children studied. In another systematic review, Horváth and Plunkett [14] found that napping seems to provide an optimal environment for consolidating memories but that daytime naps undergo changes over the course of development and become less important with brain maturity. Nevertheless, data collected from infants and toddlers are scarce and are mostly concerned with cognition. Few studies have focused on emotional consequences of napping in infants and toddlers. For example, some studies found that when toddlers are deprived of a mid-day nap, they show significantly more negative behavior and less mature self-regulation skills when faced with an unsolvable puzzle task [15] and subjectively rate emotionally salient stimuli more strongly [16]. Furthermore, it was found that an acute loss of daytime sleep in regularly napping preschoolers leads to moderate-to-large decrement in self-regulation, emotion processing, and learning [15,16,17]. Particularly, Berger and colleagues [15] conducted an experimental study of daytime sleep restriction among 10 children aged 30–36 months (seven females and three males) trained to a nap schedule (at least 5 days before emotion assessments to minimize the influence of sleep restriction ≥12.5 h time in bed per 24-h day). They compared, through videorecording, facial emotional responses in nap and no-nap conditions when viewing emotion-eliciting validated pictures (positive, negative and neutral) and completing puzzle. When sleep was restricted (no-nap condition) a reduction in positive facial emotional responses and an increase in negative facial emotional responses to pictures and puzzle-task were found. Despite the small sample size, these preliminary results suggested that when daytime sleep is insufficient, children may not manage emotion regulation challenges effectively, and this potentially places them at risk for future emotional problems.

A common practice in childcare programs is scheduling a mandatory naptime throughout childcare years [18,19]. One study longitudinally evaluated 34 children aged 5 years old (19 boys and 15 girls) with respect to sleep parameters (sleep duration, sleep efficiency and bedtime). Authors found that transition to kindergarten was associated with a decrease in weekday sleep and, despite an increased sleep pressure, an insufficient amount of total sleep time compared to the recommendations [20]. This suggested that the transition to kindergarten may challenge the sleep–wake system and this could have daytime negative effects. Previous evidence has highlighted that night sleep parameters and quality are influenced by napping practice [13]. According to Ferber [21], stopping the nap practice too early (before 3 years of age) could have negative consequences such as physical stress, fatigue and increase in night-time awakenings. A better understanding of the relationship between daytime sleep and emotions in early infancy is of utmost urgency.

### 1.3. Gap in the Literature and Aims

The key role of nocturnal sleep for healthy children’s development is increasingly documented. Instead, there is no sufficient evidence pointing out the value of prolonging napping, whether at home or in childcare contexts, once sleep has consolidated into the night. Clinical guidelines specified that napping is extremely important for cognitive functioning and attention skills but data are still lacking [22]. Despite that and despite the importance of early sleep problems in predicting later development of emotional and behavioral problems, a lack of research on daytime sleep during toddlerhood emerged. This thereby limits our knowledge, as the literature currently highlights mixed results about the influence of daytime naps on cognitive development and emotional regulation in young children [23]. The practice of napping in a childcare context is controversial. Indeed, it remains unclear if naps play a significant role in promoting learning and performance in preschoolers [24], and few studies describe sleep–wake patterns in toddlers attending nursery regularly [25,26]. Furthermore, even less is known about the importance of napping for socioemotional functioning.

Consequently, in this study, we aimed to explore the relationship between diurnal sleep habits in a nursery setting and emotions in infants and toddlers (aged 6–36 months). Specifically, the main aim of this study was to investigate the link between diurnal sleep patterns and intensity of positive and negative emotions in nursery settings, also considering the mediation role of nocturnal sleep patterns. In order to have the most accurate interpretation of the results, the secondary aim was to describe, in detail, children’s diurnal sleep habits in a nursery setting accounting for different age groups.

## 2. Materials and Methods

### 2.1. Procedure and Participants

**Figure 1:**
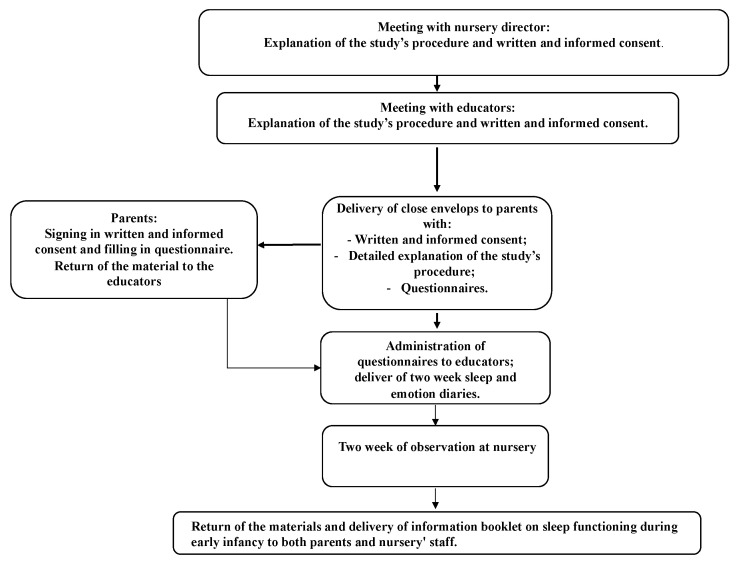
Procedure of the study.

Figure 1 shows the study flow chart. Data were collected by graduate psychology students of University of Rome Guglielmo Marconi through advertising in nursery schools as part of their bachelor thesis. At first, nursery directors were approached and, if interested, were asked to sign a detailed informed consent of the study. After that, a meeting was scheduled with educators during which students provided detailed information about the study. Written and informed consent was obtained from all educators. In this phase of the study, closed envelopes containing written and informed consent to sign in and questionnaires were handed to educators who in turn handed them to the families of the children.

Once the parent’s consent was obtained, educators received a battery of questionnaires and sleep and emotion diaries to fill in for two scholastic weeks excluding the weekend (see description of instruments below). Specifically, educators were instructed to fill in the sleep and emotion diary within 30 min after the final awakening of the children’s nap. At the end of the two weeks, participants returned the material to students.

All procedures were performed in accordance with the 1964 Helsinki Declaration and its later amendments and the study was approved by the local ethical committee of the psychological area of the University of Rome Guglielmo Marconi. Confidentiality and privacy of all participants were guaranteed through the creation of an alphanumeric code affixed to the questionnaires, which could not be paired with the name and surname of participants. These were contained in the informed consent and were kept separate from questionnaires. As reimbursement for their participation, parents and educators received an informative booklet on sleep functioning during early infancy and strategies to help children sleep.

Participants were recruited from 4 nurseries from central Italy (Rome) and northern Italy (Milan, Rovigo and Bergamo). Inclusion criteria for infants and toddlers to be recruited in the study were:

- To attend an Italian nursery school;
- To take at least one nap during nursery time;
- To be aged from 6 to 36 months;
- To not report acute or relevant physical or psychological pathologies.

No other exclusion criteria were applied to participate in the study.

### 2.2. Instruments

#### 2.2.1. For Parents

- Questionnaire about sociodemographic information

Parents were asked to complete questions about their age, work position and work satisfaction (from 0 = none at all to 4 = a lot), retrospective sleep quality before and after childbirth (from 0 = bad to 4 = good) and family organization (help by a baby sitter, grandparents and satisfaction with this help). Moreover, information about present and past psychopathologies and insomnia were requested.

- Child Behavior Checklist for Ages 1.5 to 5 [27]

The Child Behavior Checklist (CBCL) is a questionnaire composed of 99 items based on the preceding two months. It was filled in by the parent/caretaker who spends the most time with the child. In this study, the CBCL was used as an instrument for a descriptive purpose for tendencies for internalizing and externalizing problems. For this reason, we described a sample evaluating only the specific subscales “internalizing” and “externalizing” subscales and not the total score. Internalizing problems are operationalized through the following subscales: “Emotionally reactive”, “Anxious depressed”, “Somatic complaints”, “Withdrawn”; and externalizing problems are operationalized through the following subscales: “Attention problems”, “Aggressive behavior”. Scoring for identify clinical range was performed following the manual’ scoring sheet (standardized T-scores are categorized as within normal limits (T < 60); at risk (T = 0–69) and clinically significant (T ≥ 70). Nevertheless, the version of the CBCL we used is directed to children aged between 18 and 60 months, thus a bit older compared to the children of our sample. Consequently, we did not interpret the results as clinically relevant, but only as descriptive indicators of possible tendencies towards internalizing and externalizing problems. The scale was chosen, by authors’ consensus, as standardly used in pediatric research and clinical care, e.g., [28]. Parents were asked to fill in the questionnaire thinking about their child and answering as accurately as possible. Furthermore, if they had any doubts or questions, the data collection manager was instructed to always be available to answer through the telephone. Scores at CBCL were only used for a descriptive purpose and not for a diagnostic one.

- Brief Infant Sleep Questionnaire (BISQ) [29]

The BISQ is a brief, nine-item, multiple choice scale. This questionnaire includes specific questions (referring to the last week) about infant (0–36 months) daytime and night-time sleep patterns, sleep-related behavior, sleeping arrangements, bedtime rituals, and other parental interventions. The BISQ aims to evaluate sleep pattern and habits and it is composed of questions related to the following areas: (a) bedtime, (b) rise time, (c) nocturnal sleep duration, (d) daytime sleep duration, (e) number of night-time awakenings, (f) nocturnal wakefulness, (g) latency to falling asleep during the night (or settling time), (h) method of falling asleep, and (i) location of sleep.

- Sleep Disturbance Scale for Children (SDSC) [30]

This questionnaire is composed of a first part that assesses the medical story of the child from birth and a second part includes questions about children’s sleep habits and sleep disorders. The last part assesses the presence of familiarity with sleep disorders, asking who in the family had sleep disorders and what type they were. All questions refer to the past 6 months of the child’s life. Data from this questionnaire were only used for a descriptive purpose and not for a diagnostic one.

#### 2.2.2. For Educators

- Questionnaire about general sleep habits of children and emotions

For each child, educators answered questions about general sleep habits at the nursery (e.g., how the child falls asleep, with which objects and sleep onset latency) and about emotional states by filling in the Positive and Negative Affect Scale for Children (PANAS-C) [31]. This is a 27-item self-report including 12 positive emotions and 15 negative emotions. Items are scored on a 5-point Likert scale ranging from 1 (very slightly or not at all) to 5 (extremely). Educators were instructed to indicate for each child the habitual intensity of each emotion. The sample of this study was composed of younger children with respect to the reference scale but educators could contact the data collectors directly in order to solve any doubts or questions.

- Two weeks sleep and emotion diaries

The sleep diary is a standardized, self-report form that asks educators to note specific sleep-related events on a daily basis about children’s sleep in the nursery. Each day for each child, educators were instructed to answer questions about sleep within 30 min after the last awakening. From this information, it was possible to derive the total sleep time (TST) and sleep onset latency (SOL). The sleep diary is considered the gold standard among subjective measures of sleep [32]. Data were filled in for two weeks, for each day the child attended the nursery (i.e., 10 days in total, excluding the weekend). Emotions during the two weeks were assessed with the PANAS-C completed every day after the last daily nap.

### 2.3. Statistical Analysis

All statistical analyses were performed using SPSS software, version 25.0 (SPSS Inc. Chicago, IL). Before proceeding with the analyses, we excluded 8 participants because of age above 36 months, and 22 participants because they had more than 1 week of missing data; a total of 92 participants were included in the study analyses.

Descriptive and frequency analyses on the sociodemographic questionnaire, BISQ, CBCL and questionnaire about general habits of children were performed in order to analyze the sample characteristics. To describe age differences, our sample was divided into three age groups: children aged from 6 to 12 months (*n* = 17); children aged from 13 to 24 months (*n* = 44) and children aged 25 to 36 months (*n*= 31).

Using the data extracted from sleep diaries of children, the following sleep indices were calculated: diurnal SOL and diurnal TST. Moreover, positive and negative emotions were calculated from PANAS-C. Sleep parameters and positive and negative emotions were averaged over the two weeks of the study and then analyzed.

Descriptive analyses were performed also for nocturnal and diurnal sleep habits within each age group. We further conducted a multivariate analysis of variance (MANOVA) in order to compare the three groups on diurnal sleep parameters (TST and SOL) and positive and negative emotions in the nursery. Post-hoc comparisons using the Tukey HSD test were performed.

In order to investigate the relationship between diurnal sleep quality and duration and habitual and after-nap positive and negative emotions, partial correlations controlling for the effect of age (as continuous variable) and parental psychopathologies presence (as reported in the sociodemographic questionnaire) were performed.

Finally, multiple mediation models, with nocturnal sleep parameters as reported by parents (nocturnal SOL and nocturnal TST) as mediators of the effect of diurnal sleep parameters in nursery (diurnal SOL and diurnal TST) on positive emotions, controlling for age, were examined. Direct, indirect effects (specific and total), and total effects were estimated. The direct path is the effect of a variable X on a variable Y. The specific indirect effect is the path linking X to Y via a specific mediator. The total indirect effect of X on Y is the sum of the specific indirect effects of all mediators. The total effect of X on Y is the sum of the direct and indirect effects. When two or more variables are significant mediators of the relationships between X and Y, the indirect effects are significant in the mediation analyses. The bootstrap estimates were based on 5000 bootstrap samples and a 95% CI was considered. Mediation analysis was computed on PROCESS macro for SPSS [33].

## 3. Results

### 3.1. Sample Characteristics and Sleep Habits

The sample was composed of 92 mothers aged 23 to 50 years (34.68 ± 5.02 years), 92 fathers aged 23 to 55 years (38.49 ± 5.92 years) and 92 children aged 6 to 36 months (22.67 ± 8.71 months). Of these children, 52 were females, 40 were males and 56 were firstborns (see Table 1). Overall, 56% of the mothers and 58% of the fathers had a high school diploma and 20% of the mothers and 32% of the fathers had a college degree. Furthermore, eight parents reported experiencing an anxiety disorder currently or in the past, eight reported insomnia disorder and four experienced depression disorder. Results on CBCL scores (that were all compiled by mothers) showed that eight children presented clinical internalizing problems, and three children presented clinical externalizing problems. Sample characteristics are summarized in Table 1.

Descriptive analysis on the BISQ showed nocturnal sleep ranging from 5 to 12 h (on average 9.04 ± 1.13 h), wake after nocturnal sleep (WASO) ranging from 0 to 180 min (on average 28.26 ± 41.69 min) and a sleep onset latency (SOL) ranging from 0 to 150 min (on average 28.24 ± 23.55 min). Furthermore, parents reported that 31 children sleep in a separate room, eleven in a room with their brothers and sisters, 33 in the same room as the parents, 15 in the same bed as the parents and 2 in other places. In addition, only 38 children fell asleep alone in their bed and 54 fell asleep in other places only to be put to bed at a later time.

Results reported by parents on BISQ for age groups are reported in Table 2 and showed that children aged 25 to 36 months had longer sleep onset latencies compared to other age groups. Furthermore, a significant difference between males and females of this age group was found in SOL (F = 12.09, *p* < 0.005), indicating longer SOL in males. Diurnal sleep hours decreased across age groups, except for females aged 25 to 36 months. Regarding nocturnal sleep, every age group was reported to sleep less than the recommended number of hours by the National Sleep Foundation [34], as shown in Table 2. Females aged 6 to 12 months and males aged 25 to 36 months had the shortest nocturnal sleep duration compared to other groups.

Children of our sample slept at nursery one to three times per day; 63% of them were reported to be able to fall asleep alone, 29% with the proximity of an adult, 7% needed to be held by an adult and only three of them were defined by educators as being very resistant to falling asleep. Furthermore, 87% of the children usually woke up quietly and 13% crying and complaining. Educators reported that only 25 children took a long time to fall asleep. On average, children in nursery slept 95.75 ± 32.82 min, and had a sleep onset latency of 9.98 ± 32.82 min. Children, during nursery time, present on average a score of 3.16 ± 0.67 on positive emotions and of 1.51 ± 0.45 on negative emotions.

### 3.2. Differences between Children of Different Age in Intensity of Positive and Negative Emotions and Sleep Pattern in the Nursery

Multivariate analysis of variance (MANOVA) was performed in order to compare the three age groups on diurnal sleep parameters (TST and SOL as rated by educators at nurseries) and positive and negative emotions in the nursery (rated by educator at nurseries). Results showed a significant effect of the group on diurnal TST (F _(1;89)_ = 12.66, *p* ≤ 0.001) and diurnal SOL (F _(1;89)_ = 6.22, *p* ≤ 0.005). Post-hoc comparisons using the Tukey HSD test indicated that the mean diurnal TST in children aged 6 to 12 months (64.18 ± 1.30 min) was significantly lower than those of children aged 13 to 24 months (103.28 ± 29.97 min) and of children aged 25 to 36 months (103.89 ± 23.61 min). Moreover, the mean diurnal SOL times in children aged 25 to 36 months (12.58 ± 6.03 min) and in children aged 13 to 24 months (8.42 ± 4.80 min) were significantly different from children aged 6 to 12 months (8.94 ± 4.27 min). However, no significant differences were found for positive and negative emotion scores in the three age groups.

### 3.3. Diurnal Sleep and Intensity of Positive and Negative Emotions in Nursery Settings and the Mediation Role of Nocturnal Sleep Patterns

Partial correlation analyses (two-tail) were performed, controlling for the effect of the age of children (as continuous variable) and parental psychopathologies presence, and showed a significant negative correlation between habitual positive emotions (as reported by educators in the sociodemographic questionnaire) and diurnal SOL (r = −0.36, *p* < 0.001).

Results of partial correlation analysis (two-tail), controlling for the effect of the age of children and parental psychopathologies presence, on sleep parameters and post-nap positive and negative emotions are reported in Table 3. Results showed a significant negative correlation between diurnal SOL and positive emotions (r = −0.30, *p* < 0.005) and a significant positive correlation between diurnal total sleep time and positive emotions (r = −0.22, *p* = 0.03) No significant correlations were found for negative emotions.

Two mediation models were tested. The first one tested the mediation role of nocturnal SOL and nocturnal TST in the relationship between diurnal SOL and positive emotions. The total effect of diurnal SOL on positive emotions intensity (*t* = −3.45, *p* < 0.05) was significant, but the direct effect of nocturnal parameters on positive emotions (nocturnal SOL: *t* = 0.11, *p* = 0.90; nocturnal TTS: *t* = −0.45, *p* = 0.64) was not. The second model tested the mediation role of nocturnal sleep parameters (nocturnal SOL and nocturnal TST) in the relationship between diurnal TST and positive emotions. The total effect of diurnal TST on positive emotions intensity was not significant (*t* = 0.89, *p* = 0.37) and the direct effect of nocturnal parameters were also not significant (nocturnal SOL: *t* = 0.19, *p* = 0.84; nocturnal TTS: *t* = −0.71, *p* = 0.47).

## 4. Discussion

This study aimed at investigating toddler’s sleep habits during nursery time and the relationship between sleep patterns and intensity of positive and negative emotions. Results showed that children of our sample slept on average one hour and a half with average sleep onset latency of ten minutes. Moreover, they generally had good sleep quality and good sleep hygiene habits (e.g., self-soothing ability). Another interesting result, consistent with previous findings [5,24], was that parent-reported diurnal sleep of children in our sample decreased across age. Furthermore, it is well-known that the development and the consolidation of regular sleep–wake patterns in early childhood is a process that changes rapidly and keeps evolving throughout childhood [35]. Results on different age groups showed that children aged 6 to 12 months had significantly less total sleep time at nursery as reported by educators compared to children aged 13 to 24 months and children aged 25 to 36 months. These results are in contrast to previous findings [5,24] and to information about sleep reported by parents. This could be due to the fact that subjective reports of parents are biased by the tendency to underestimate variables of sleep quality [26,36]. However, although differences in sleep patterns were found, results showed no significant differences for the intensity of positive and negative emotions across the three age groups. This could be due to the low accuracy of educators in rating children’s negative and positive emotions, e.g., [37].

Our main objective was to investigate the relationship between diurnal sleep in nursery and habitual and post-nap positive and negative emotions. Results showed a significant positive relationship between total sleep time and post-nap positive emotions, and a significant negative relationship between sleep onset latency and post-nap positive emotions. These results are consistent with previous results about nocturnal sleep patterns and positive and negative emotions and the ability to regulate emotions [38] but less is known about this relationship in a nursery context, specifically for diurnal sleep patterns and post-nap emotions. Previous findings had pointed out the importance of positive and negative emotions in sleep disorders [39] but few studies investigated this relationship in young children and considering diurnal sleep. Since sleep problems in the pediatric population are associated with behavioral and emotional issues (such as depression and anxiety symptomatology, separation distress, and inhibition) in the course of the development [11], future studies should investigate this association further with longitudinal designs in order to clarify the direction of this association. Specifically, future longitudinal studies could deepen the understanding of the role and the importance of naps by observing children’s sleep patterns at different stages of development such as at nursery, kindergarten, primary school and secondary school, considering both home and school environments. Moreover, future studies should combine subjective and physiological measures to assess both emotional and sleep aspects.

Furthermore, positive emotions seem to be linked to diurnal sleep aspects, confirming previous result [15], thus the importance of napping in childcare contexts should be reinforced in educational programs. Additionally, results of the mediation analysis revealed that the effect of the diurnal sleep patterns on positive emotions was not mediated by nocturnal sleep patterns in our sample, evidencing that daytime napping is relevant for emotional functioning independent of nocturnal sleep quality.

One strength of this study is that it is one of the few studies investigating the relationship between sleep patterns and emotions in nursery time; moreover, educators rated sleep and emotional patterns of children at the moment after waking and not retrospectively. Findings of this study could have some important clinical and practical implications. For example, intervention studies are needed to examine whether sleep programs for early childhood may prevent later emotional and behavioral outcomes. Furthermore, since a connection between diurnal sleep and emotions seems to exist in early childhood, ecological momentary assessment and longitudinal studies on sleep patterns and emotion regulation in this age group are needed to better understand the direction of this association and to perform effective sleep programs in childcare.

Our study presents some limitations. At first, the variability across the different nurseries (e.g., nap-time, environment, educators’ involvement) in napping habits should be considered in order to understand the most positive characteristics for an optimal nap schedule. Furthermore, at home, sleep environment and daily routines may influence the results both on daytime sleep pattern and on the intensity of positive and negative emotions, e.g., [40]. Despite that, our result showed that nocturnal sleep patterns were not mediators of the relationship between diurnal sleep and emotions. This could be due to different instruments used for assessing nocturnal sleep patterns (assessed by parents with standardized questionnaires) and diurnal sleep patterns (assessed by educators at nursery with sleep diaries). Future studies should investigate further this relationship using consistent instruments that could be compared. Furthermore, different sleep schedules and nap-time in nurseries and kindergartens could be further investigated to provide detailed guidelines that could be followed in a school context to warrant an adequate amount of sleep and promote health sleep habits in children.

Moreover, a selection bias could occur since the nurseries were a convenience sample selected by contacts and in the proximity of the data collectors and the recruited sample was small and not representative of the population (since children who did not nap are not included). Moreover, one of the eligibility criteria of our study was to include only children who take at least one nap in the nursery, so we did not consider the intensity of emotions and sleep pattern of children who did not sleep in the nursery. A further investigation of the differences in sleep and emotional patterns between these two populations should be performed. Finally, only subjective measures, and not specific for this age range were used and cross—sectional design was performed. Future studies should deepen the understanding of the relationship between sleep patterns, sleep quality and positive and negative emotional intensity in childcare, selecting nurseries with similar nap schedules and that are more representative, and using objective measures (e.g., actigraphy) and longitudinal or ecological momentary assessment design.

## 5. Conclusions

To conclude, there is evidence that stresses the importance of regular diurnal sleep patterns in infants and toddlers for a healthy development. It appears to be of utmost importance to evaluate the connection between not only nocturnal sleep but also diurnal sleep patterns and habits and the management of positive and negative emotions in preschoolers. A better understanding of these processes might help to advance clinical practice. To understand what kind of behavior, habit or pattern is unhealthy or dysfunctional could lead to providing effective preventive intervention in the pediatric population and education from pediatric primary care.

## Figures and Tables

**Table 1:** Sample Characteristics.

	Mothers (*n* = 92)	Fathers (*n* = 92)	Children (*n* = 92)
Demographics	(Mean ± SD)	(Mean ± SD)	(Mean ± SD)
Age	34.68 ± 5.02	38.49 ± 5.92	22.67 ± 8.71
Education level	Middle school: 11% High school: 56% College: 32% Advanced education: 1%	Primary school: 1% Middle school: 18% High school: 58% College: 20% Advanced education: 1%	
Co-morbid conditions	*n*	*n*	*n*
Depression	2	2	n.a.
Anxiety	5	3	n.a.
Insomnia	3	5	n.a.
Externalizing problems	n.a.	n.a.	3
Internalizing problems	n.a.	n.a.	8

Age of parents = in years; age of children = in months; middle school = education between primary school and secondary school; co-morbid conditions for parents = self-reported in sociodemographic questionnaire reporting “yes” or “no” for each reported condition; co-morbid conditions for children = clinical range scoring reported by parents in Child Behavior Checklist (CBCL); n.a.= not applicable.

**Table 2:** Children’s sleep habits as reported by parents.

	Children Aged 6–12 Months (*n* = 17)		Children Aged 13–24 Months (*n* = 44)		Children Aged 25–36 Months (*n* = 31)		Overall (*n* = 92)	
	Males	Females	Males	Females	Males	Females	Males	Females
	*n* = 5	*n* = 12	*n* = 23	*n* = 21	*n* = 12	*n* = 19	*n* = 40	*n* = 52
Nocturnal sleep (Hours) Mean ± SD	9.70 ± 1.09	8.66 ± 1.55	9.17 ± 0.74	9.52 ± 0.86	8.54 ± 1.67	8.77 ± 0.89	9.05 ± 1.17	9.04 ± 1.12
Diurnal sleep (Hours) Mean ± SD	2.60 ± 0.54	2.48 ± 1.37	2.29 ± 0.75	2.07 ± 0.59	1.83 ± 0.61	2.09 ± 0.65	2.19 ± 0.72	2.18 ± 0.87
Sleep onset latency (Minutes) Mean ± SD	20.00 ± 16.83	20.83 ± 15.49	31.09 ± 19.18	20.24 ± 15.30	45.17 ± 45.99	* 26.42 ± 14.79	33.93 ± 29.69	22.83 ± 15.11
Wake after sleep onset (Minutes) Mean ± SD	20.00 ± 9.35	26.36 ± 36.40	31.71± 42.67	18.57 ± 22.67	37.50 ± 60.82	29.16 ± 49.99	31.69 ± 44.84	25.66 ± 39.10

* Statistically significant difference between males and females of 25–36 months group: F = 12.09, *p* < 0.005.

**Table 3:** Correlation between sleep parameters and post-nap positive and negative emotions.

		Positive Emotions	Negative Emotions
Diurnal total sleep time	r	0.229	−0.150
	Sign.	0.030	0.158
	df	89.00	89.00
Diurnal sleep onset latency	r	−0.309	−0.180
	Sign.	0.003	0.089
	df	89.00	89.00

Total sleep time; sleep onset latency; positive and negative emotions = averaged across the two weeks of diaries; df: degree of freedom.

## References

[1] Bathory E., Tomopoulos S. (2017). Sleep regulation, physiology and development, sleep duration and patterns, and sleep hygiene in infants, toddlers, and preschool-age children. Curr. Probl. Pediatr. Adolesc. Health Care.

[2] Figueiredo B., Dias C.C., Pinto T.M., Field T. (2016). Infant sleep-wake behaviors at two weeks, three and six months. Infant Behav. Dev..

[3] De Roquefeuil G., Djakovic M., Montagner H. (1993). New data on the ontogeny of the child’s sleep-wake rhythm. Chronobiol. Int..

[4] Weissbluth M. (1995). Naps in children: 6 months–7 years. Sleep.

[5] Iglowstein I., Jenni O.G., Molinari L., Largo R.H. (2003). Sleep duration from infancy to adolescence: Reference values and generational trends. Pediatrics.

[6] Hatzinger M., Brand S., Perren S., Von Wyl A., Stadelmann S., Von Klitzing K., Holsboer-Trachsler E. (2014). In pre-school children, sleep objectively assessed via sleep-EEGs remains stable over 12 months and is related to psychological functioning, but not to cortisol secretion. J. Psychiatr. Res..

[7] Lam J.C., Mahone E.M., Mason T.B.A., Scharf S.M. (2011). The effects of napping on cognitive function in preschoolers. J. Dev. Behav. Pediatr..

[8] Vaughn B.E., Elmore-Staton L., Shin N., El-Sheikh M. (2015). Sleep as a support for social competence, peer relations, and cognitive functioning in preschool children. Behav. Sleep Med..

[9] Ann Easterbrooks M., Biringen Z. (2000). Guest editors’ introduction to the special issue: Mapping the terrain of emotional availability and attachment. Attach. Hum. Dev..

[10] Hysing M., Siversten B., Garthus-Niegel S., Eberhard-Gran M. (2016). Pediatric sleep problems and social-emotional problems. A popu- lation-based study. Infant Behav. Dev..

[11] Mindell J.A., Leichman E.S., DuMond C., Sadeh A. (2017). Sleep and social-emotional development in infants and toddlers. J. Clin. Child Adolesc. Psychol..

[12] Sadeh A., Gruber R., Raviv A. (2002). Sleep, neurobehavioral functioning, and behavior problems in school-age children. Child Dev..

[13] Thorpe K., Staton S., Sawyer E., Pattinson C., Haden C., Smith S. (2015). Napping, development and health from 0 to 5 years: A systematic review. Arch. Dis. Child.

[14] Horváth K., Plunkett K. (2018). Spotlight on daytime napping during early childhood. Nat. Sci. Sleep.

[15] Berger R.H., Miller A.L., Seifer R., Cares S.R., LeBourgeois M.K. (2012). Acute sleep restriction effects on emotion responses in 30- to 36-month old children. J. Sleep Res..

[16] Miller A.L., Seifer R., Crossin R., LeBourgeois M.K. (2015). Toddler’s self-regulation strategies in a challenge context are nap-dependent. J. Sleep Res..

[17] Kurdziel L., Duclos K., Spencer R.M. (2013). Sleep spindles in midday naps enhance learning in preschool children. Proc. Natl. Acad. Sci. USA.

[18] Komada Y., Asaoka S., Abe T., Matsuura N., Kagimura T., Shirakawa S., Inoue Y. (2012). Relationship between napping pattern and nocturnal sleep among Japanese nursery school children. Sleep Med..

[19] Tayler C., Ishimine K., Cloney D., Cleveland G., Thorpe K. (2013). The quality of early childhood education and care services in Australia. Australas. J. Early Child.

[20] Cairns A., Harsh J. (2014). Changes in sleep duration, timing, and quality as children transition to kindergarten. Behav. Sleep Med..

[21] Ferber R., Ferber R., Kryger M. (1995). Assessment of sleep disorders in the child. Principles and Practice of Sleep Medicine in the Child.

[22] Staton S., Rankin P.S., Harding M., Smith S.S., Westwood E., LeBourgeois M.K., Thorpe K.J. (2020). Many naps, one nap, none: A systematic review and meta-analysis of napping patterns in children 0–12 years. Sleep Med. Rev..

[23] Mindell J.A., Owens J.A. (2015). A Clinical Guide to Pediatric Sleep: Diagnosis and Management of Sleep Problems.

[24] Lam J.C., Koriakin T.A., Scharf S.M., Mason T.B., Mahone E.M. (2019). Does increased consolidated nighttime sleep facilitate attentional control? A pilot study of nap restriction in preschoolers. J. Atten. Dis..

[25] Acebo C., Sadeh A., Seifer R., Tzischinsky O., Hafer A., Carskadon M.A. (2005). Sleep/wake patterns derived from activity monitoring and maternal report for healthy 1- to 5-year-old children. Sleep.

[26] Tikotzky L., Sadeh A. (2001). Sleep patterns and sleep disruptions in kindergarten children. J. Clin. Psychol..

[27] Achenbach T.M., Rescorla L.A. Child Behavior Checklist for Ages 1 1/2-55. https://aseba.org/wp-content/uploads/2019/02/preschoolcbcl.pdf.

[28] Maltby L.E., Callahan K.L., Friedlander S., Shetgiri R. (2019). Infant temperament and behavioral problems: Analysis of high-risk infants in child welfare. J. Public Child Welf..

[29] Sadeh A. (2004). A brief screening questionnaire for infant sleep problems: Validation and findings for an Internet sample. Pediatrics.

[30] Bruni O., Ottaviano S., Guidetti V., Romoli M., Innocenzi M., Cortesi F., Giannotti F. (1996). The Sleep Disturbance Scale for Children (SDSC) Construction and validation of an instrument to evaluate sleep disturbances in childhood and adolescence. J. Sleep Res..

[31] Laurent J., Catanzaro S., Joiner T., Rudolf K., Potter K., Lambert S. (1999). A measure of positive and negative affect for children: Scale development and preliminary validation. Psychol. Assess..

[32] Buysse D.J., Ancoli-Israel S., Edinger J.D., Lichstein K.L., Morin C.M. (2006). Recommendations for a standard research assessment of insomnia. Sleep.

[33] Hayes A.F., Preacher K.J., Hancock G.R., Mueller R.O. (2013). Conditional process modeling: Using structural equation modeling to examine contingent causal processes. Quantitative Methods in Education and the Behavioral Sciences: Issues, Research, and Teaching. Structural Equation Modeling: A Second Course.

[34] Hirshkowitz M., Whiton K., Albert S.M., Alessi C., Bruni O., DonCarlos L., Hazen N., Herman J., Katz E.S., Kheirandish-Gozal L. (2015). National Sleep Foundation’s sleep time duration recommendations: Methodology and results summary. Sleep Health.

[35] Sadeh A.V.I., Mindell J.A., Luedtke K., Wiegand B. (2009). Sleep and sleep ecology in the first 3 years: A web-based study. J. Sleep Res..

[36] Sadeh A., Raviv A., Gruber R. (2000). Sleep patterns and sleep disruptions in school-age children. Dev. Psychol..

[37] Karing C., Dörfler T., Artelt C. (2015). How accurate are teacher and parent judgements of lower secondary school children’s test anxiety?. Educ. Psychol..

[38] Palmer C.A., Alfano C.A. (2017). Sleep and emotion regulation: An organizing, integrative review. Sleep Med. Rev..

[39] Baglioni C., Spiegelhalder K., Lombardo C., Riemann D. (2010). Sleep and emotions: A focus on insomnia. Sleep Med. Rev..

[40] Ward T.M., Gay C., Anders T.F., Alkon A., Lee K.A. (2007). Sleep and napping patterns in 3-to-5-year old children attending full-day childcare centers. J. Pediatr. Psychol..

